# The Difference of Volatile Compounds in Female and Male Buds of *Herpetospermum pedunculosum* Based on HS-SPME-GC-MS and Multivariate Statistical Analysis

**DOI:** 10.3390/molecules27041288

**Published:** 2022-02-14

**Authors:** Zhenying Liu, Ye Fang, Cui Wu, Xian Hai, Bo Xu, Zhuojun Li, Pingping Song, Huijun Wang, Zhimao Chao

**Affiliations:** 1 Institute of Chinese Materia Medica, China Academy of Chinese Medical Sciences, Beijing 100700, China; liuzy9607@163.com (Z.L.); wucuidalian@163.com (C.W.); xubo_345@163.com (B.X.); 18811385399@163.com (Z.L.); songpingping122@163.com (P.S.); huijunde163@163.com (H.W.); 2 Shangri-La Alpine Botanical Garden, Diqing 674400, China; sabg001@163.com (Y.F.); haixian9310@126.com (X.H.)

**Keywords:** *Herpetospermum pedunculosum*, HS-SPME-GC-MS, dioecious plant, bud, volatile compound, multivariate statistical analysis

## Abstract

*Herpetospermum pedunculosum* (Ser.) C. B. Clarke (Family Cucurbitaceae) is a dioecious plant and has been used as a traditional Tibetan medicine for the treatment of hepatobiliary diseases. The component, content, and difference in volatile compounds in the female and male buds of *H. pedunculosum* were explored by using headspace solid-phase microextraction-gas chromatography-mass spectrometry (HS-SPME-GC-MS) technology and multivariate statistical analysis. The results showed that isoamyl alcohol was the main compound in both female and male buds and its content in males was higher than that in females; 18 compounds were identified in female buds including 6 unique compounds such as (*E*)-4-hexenol and isoamyl acetate, and 32 compounds were identified in male buds, including 20 unique compounds such as (*Z*)-3-methylbutyraldehyde oxime and benzyl alcohol. (*Z*)-3-methylbutyraldehyde oxime and (*E*)-3-methylbutyraldehyde oxime were found in male buds, which only occurred in night-flowering plants. In total, 9 differential volatile compounds between female and male buds were screened out, including isoamyl alcohol, (*Z*)-3-methylbutanal oxime, and 1-nitropentane based on multivariate statistical analysis such as principal component analysis (PCA) and orthogonal partial least squares discrimination analysis (OPLS-DA). This is the first time to report the volatile components of *H. pedunculosum*, which not only find characteristic difference between female and male buds, but also point out the correlation between volatile compounds, floral odor, and plant physiology. This study enriches the basic theory of dioecious plants and has guiding significance for the production and development of *H. pedunculosum* germplasm resources.

## 1. Introduction

*Herpetospermum pedunculosum* (Ser.) C. B. Clarke (Family Cucurbitaceae) is an annual climbing herb, distributed in Tibet, Yunnan, Sichuan, and other high-altitude areas in China, Nepal, and northeastern India [1,2,3]. As a traditional Tibetan medicine, it has the functions of clearing away heat and detoxification, and removing the gallbladder and internal heat for its bitter taste and cool potency [3,4], and is widely used for the treatment of hepatobiliary diseases [5].

**Figure 1:**
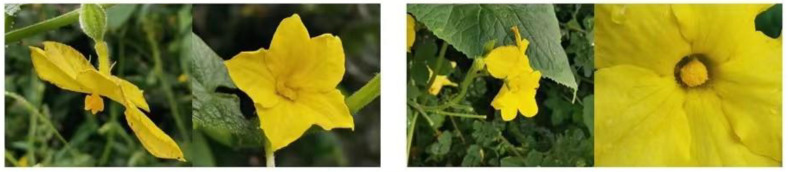
The flowers of *H. pedunculosum* (**Left**: female; **right**: male).

*H. pedunculosum* is a dioecious plant, whose bisexual flower is shown in Figure 1. Its flowering period is June to September, and it blooms at night. Its flowers are golden-yellow with five petals, trumpet-shaped, eventually tubular, and covered with fine hairs. The male flower is usually solitary with sparsely pubescent hairs or concomitant with the same raceme; pedicels with 2.0–6.0 cm length and sparse villous hairs; calyx tube with 2.0–2.5 cm length, enlarged similarly to a funnel, tubular lower part, and lanceolate lobes; yellow corolla with 2.0–2.2 cm in length and 1.2–1.3 cm in width, and with elliptic lobes and sharp tips. The female flower is solitary with the same perianth as male flowers and with three staminodes or none, and oblong ovary with three rooms. The mature fruit is 7–8 cm in length and 3–4 cm in width with long pubescent hairs.

There were many studies of *H. pedunculosum*. Its seeds had some pharmacological activities of hepatoprotection, anti-hepatitis B virus, and anti-liver fibrosis [6,7], contained chemical constitutes of fatty acid, polysaccharides, coumarin, and spinasterol glycoside [6,7,8,9], and had 156,531 bp length of the whole genome [10]. Because the seeds have medicinal, economic, and sowing values, the female plant is more popular with farmers [11,12]. Fifty metabolites including carbohydrates, amino acids, organic acids, lipids, and polyamine were identified in its leaves [13]. Twelve compounds including *n*-benzyltyramine, 1H-indol-3-carboxylic acid, rhodiocyanoside B, and matteflavoside A were isolated from its stems [14]. The moisture, total ash, acid-insoluble ash, and alcohol-soluble extract were determined as quality indexes of its flower [15,16,17]. However, there were a few studies on the gender difference in the dioecious plant of *H. pedunculosum*.

In this study, the female and male buds of *H. pedunculosum* were collected. Headspace solid-phase microextraction (HS-SPME) was used to extract their volatile components. Gas chromatography-mass spectrometry (GC-MS) was used to separate and identify these volatile compounds. The multivariate statistical analysis was chosen to screen the differential compounds, aiming to explore the difference in volatile compounds between female and male buds of this dioecious plant.

## 2. Results

### 2.1. Volatile Compounds of GC-MS Analysis

**Figure 2:**
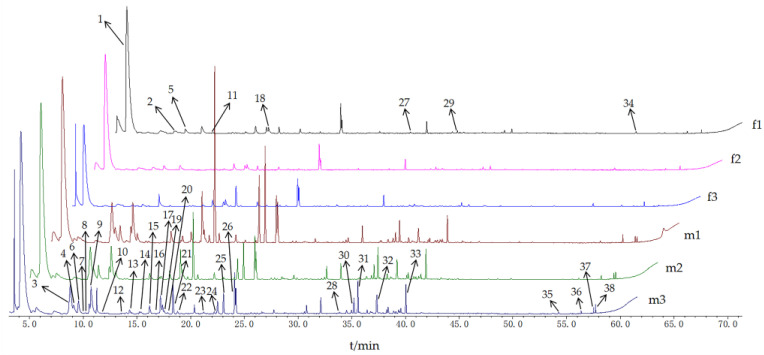
GC-MS total ion chromatogram of volatile components from female and male buds of *H. pedunculosum.*

The total ion chromatogram obtained by GC-MS analysis was shown in Figure 2. The results showed that there were no significant differences among the three female buds (f1, f2, and f3) nor among the three male buds (m1, m2, and m3), but there were significant differences between female and male buds. It was indicated that there were no significant differences of buds in the same sex, but there were essential differences between different sexes regarding their volatile constitutes.

The results of volatile compounds by GC-MS analysis were shown in Table 1. The volatile compounds were identified by comparing retention time and mass spectra. For example, compound 1 (4.044 min) yielded a parent ion at *m*/*z* 88 and fragment ions at *m*/*z* 77, 55, and 42, and was identified as isoamyl alcohol according to NIST database. Compound 3 (8.675 min) displayed a parent ion at *m*/*z* 101 and fragment ions at *m*/*z* 86, 59, and 41, and was identified as (*Z*)-3-methylbutanal oxime. Compound 9 (10.578 min) produced a parent ion at *m*/*z* 117 and fragment ions at *m*/*z* 71, 43, and 29, and was identified as 1-nitropentane.

As a result, in total, 38 volatile compounds were identified from the female and male buds of *H. pedunculosum*, including 18 compounds in female buds and 32 compounds in male buds, covering alkanes, alkenes, alcohols, aldehydes, ketones, and oximes, etc. The female and male buds had 12 common compounds, the female buds had 6 unique compounds, and the male buds had 20 unique compounds.

A compound of the highest content in volatile components from both female and male buds of *H. pedunculosum* was isoamyl alcohol whose content was 83.29%, 90.17%, and 90.19% in female buds, and was 43.23%, 57.90%, and 64.01% in male buds, and the average relative content in female buds was 1.60 times higher than that in male buds. Isoamyl alcohol has the aroma of apple brandy, which has the effect of increasing the aroma and obvious spicy taste, so its higher content may be the main reason for the weaker fragrance of female flowers and the stronger one of male flowers. Among other common compounds, the content of β-ocimene in male buds (5.58%, 5.22%, and 5.69%) was higher than that in female buds (0.56%, 0.79%, and 1.19%) and the average content in female buds was 6.47 times higher than that in male buds. The content of *δ*-limonene in male buds (2.10%, 2.95%, and 4.20%) was also higher than that in female buds (1.19%, 1.64%, and 1.95%), and the average content in male buds was 1.94 times higher than that in female buds.

The characteristic volatile compounds in female buds were (*E*)-4-hexenol, isoamyl acetate, 2-ethylhexanol, *n*-pentadecane, *n*-hexadecane, and neophytadiene. The characteristic volatile compounds in male buds had 20 compounds, including 2 oximes, 3 terpenes (β-myrcene, *α*-pinene, and α-copaene), and 4 phenyl compounds (*p*-xylene, *o*-xylene, benzaldehyde, and benzyl alcohol).

### 2.2. Multivariate Statistical Analysis

**Figure 3:**
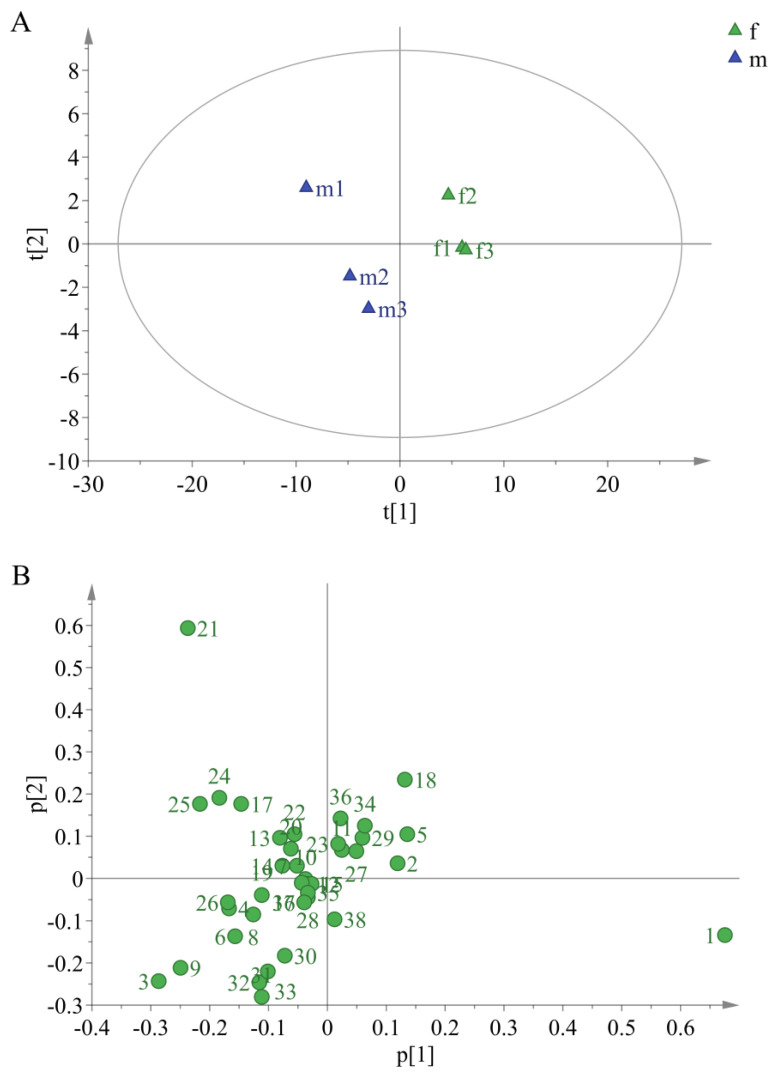
PCA scores (**A**) and Loadings (**B**) for female and male buds of *H. pedunculosum.* The green numbers in figure B were consistent with the No. in Table 1.

In order to further explore the difference of volatile compounds between female and male buds, multivariate statistical analysis such as principal component analysis (PCA) and orthogonal partial least squares discrimination analysis (OPLS-DA) were carried out. The PCA was performed on the female and male buds and the result was shown in Figure 3A. The contribution rates of principal component 1 (PC1) and principal component 2 (PC2) were 88.20% and 9.54%, respectively, and the total contribution rate was 97.74%. R^2^X and Q^2^ were 0.977 and 0.917, respectively, where R^2^X represented the fitting degree and Q^2^ represented the prediction degree of the model. Additionally, it was indicated that the model was stable and reliable from these data. The classification results of the female and male buds were ideal, the female buds were distributed on the right side of the Y axis, and the male buds were distributed on the left side of the Y axis, which suggested that the volatile compounds of different sexes were significantly different. The Loadings analysis was shown in Figure 3B. The volatile compounds with the largest contribution rate to distinguishing the female and male buds were screened with the coordinate axis 0.2 or −0.2 as the limit value. It was found that the five compounds had the largest contribution rate, i.e., isoamyl alcohol (1), 4-oxoisophorone (25), β-ocimene (21), (*Z*)-3-methylbutyraldehyde oxime (3), and 1-nitropentane (9).

**Figure 4:**
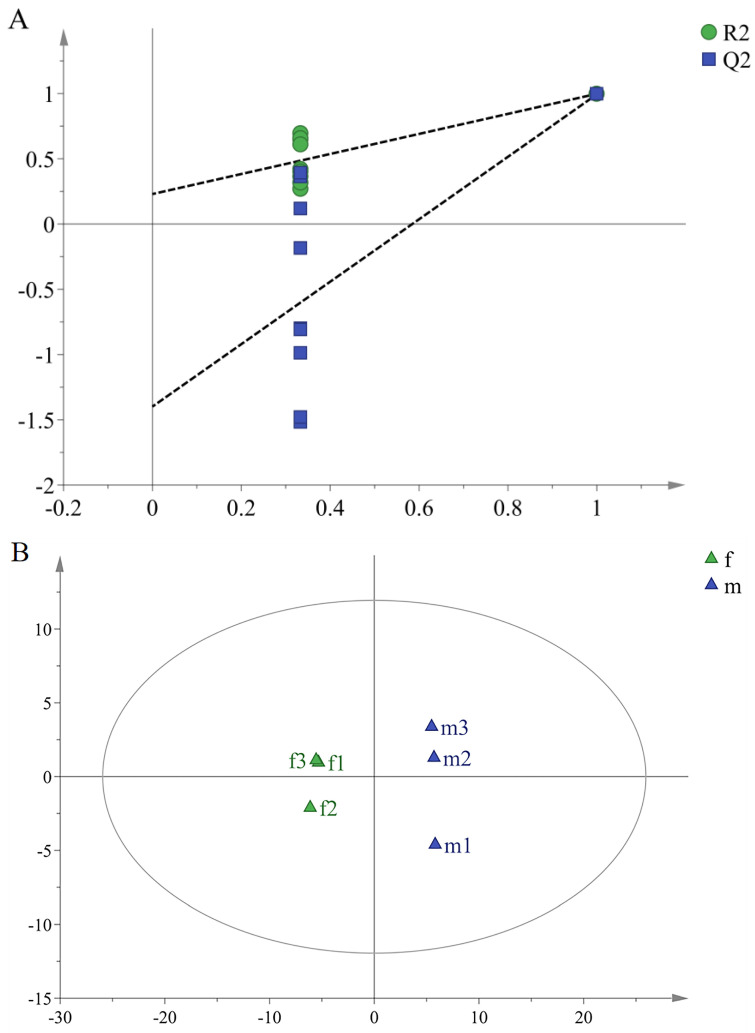

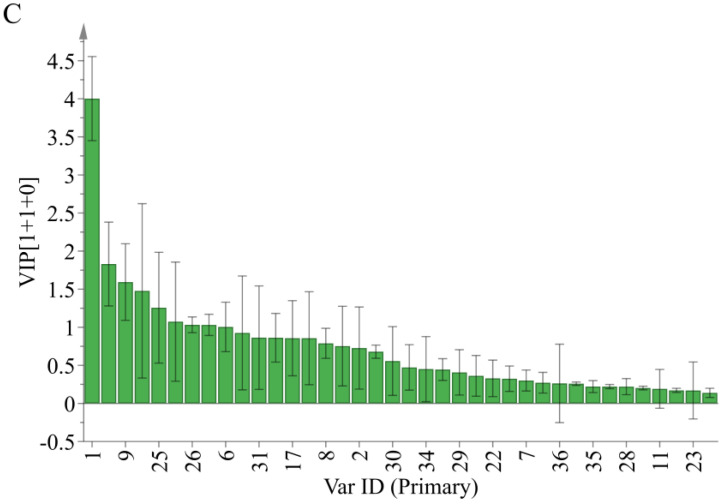
OPLS-DA arranges the model verification diagram (**A**), scores (**B**), and VIP scores (**C**) between female and male buds of *H. pedunculosum*. The numbers of Var ID in Figure C were consistent with the No. in Table 1.

As an unsupervised analysis method, PCA cannot ignore within-group and eliminate irrelevant random errors. Therefore, supervised OPLS-DA was used to further determine the difference of volatile compounds between female and male buds. However, overfitting was easy to occur while expanding the differences between groups, so it was necessary to arrange experiments with the help of external model validation methods to prove the validity of the model. Different random Q^2^ values were obtained by changing the arrangement order of the categorical variable y randomly multiple times (*n* = 200). In this experiment, the greater the slope of the regression line, the smaller the difference between R^2^ and Q^2^, indicating that there were more data with which to explain the model; the predictive ability of the model was better. The test result was shown in Figure 4A. Among them, the R^2^ and Q^2^ values generated by any random arrangement on the left end were smaller than those on the right, the slope of the regression line was large, and the lower regression line intersected the negative half-axis of the Y-axis, indicating that the model was effective, stable, and predictable, and could continue to screen the difference compounds. From the OPLS-DA scatter plot of female and male buds (R^2^X: 0.977, R^2^Y: 0.998, and Q^2^: 0.994) (Figure 4B), it could be seen that the two types of samples were clearly distinguished. According to the corresponding VIP value (Figure 4C), these variables whose VIP value was greater than 1 and whose confidence interval did not contain 0 were screened, and it was determined that there were nine differential volatile compounds between the female and male buds (Table 2). Among them were section A for isoamyl alcohol of higher levels in female buds and section B for (*Z*)-3-methylbutyraldehyde oxime, 1-nitropentane, β-ocimene, 4-oxoisophorone, 2-methyl-1-undecene, 2,2,6-trimethyl-1,4-cyclohexanedione, *p*-xylene, and (*E*)-3-methylbutyraldehyde oxime of higher levels in male buds.

## 3. Discussion

There were a lot of differences in the morphological structure between female and male flowers and buds of the dioecious plant *H. pedunculosum*, but the difference in volatile compounds has not been reported in the literature. Buds are the state when flowers are about to bloom; their chemical components have not been lost and polluted by foreign substances, thus the research on chemical components of buds has a more realistic effect. Therefore, there were many studies which took buds as research objects, such as *Eugenia caryophyllus* (Myrtaceae) [18,19,20], *Capparis spinosa* (Capparaceae) [21], *Magnolia biondii* and *M. sirindhorniae* (Magnoliaceae) [22,23], *Populus tomentosa* (Salicaceae) [24], and *Pyrus pyrifolia* (Rosaceae) [25]. In this study, it was found that there was significant difference in the volatile compounds between female and male buds of *H. pedunculosum*.

Isoamyl alcohol is a fragrance ingredient used in cosmetics, detergents, fine fragrances, household cleaners, shampoos, toilet soaps, and other toiletries [26]. Because of its advantages of high energy density, low hygroscopicity, and compatibility with the current infrastructure, isoamyl alcohol has attracted considerable attention as one of the biofuels [27]. As a result, it was showed that the content (up to 90.19%) of isoamyl alcohol was the highest, which provided a new perspective for improving the economic value of *H. pedunculosum*.

Some unique compounds in female or male buds made this study more meaningful. Two kinds of oximes and phenyl compounds were only present in male buds. For (*Z*)-3-methylbutyraldehyde oxime and (*E*)-3-methylbutyraldehyde oxime, the reports on plant volatile compounds were extremely rare: only the night-flowering plants of *Gaura drummondii* (Onagraceae) and *Trichosanthes kirilowii* (Cucurbitaceae) [28,29]. For phenyl compounds, benzaldehyde was only detected in male flowers and the content of benzyl alcohol in male flowers was significantly higher than that in female flowers of *T. kirilowii* [29]. Similarly, 4-oxoisophorone was detected only in male buds in this study, which was one of nine differential compounds filtered by multivariate statistical analysis. It was shown that 4-oxoisophorone may be a key compound for honeybees’ perceptions of flower odor, which was likely to be implicated in bee foraging behavior [30].

(*E*)-4-Hexenol, isoamyl acetate, and 2-ethylhexanol were only detected in female buds. It was found that the volatile compounds such as isoamyl alcohol and isoamyl acetate were more attractive to pollinators in the field behavior measurements [31]. High contents of alcohol and ester compounds were beneficial to better pollination of the female flowers of *H. pedunculosum*, which could promote the yield of fruits and improve the economic value of female plants.

In addition, seven terpenes were detected in female or male buds, including β-myrcene, *α*-pinene, *α*-copaene, sabinene, β-ocimene, δ-limonene, and γ-terpinene. It was shown that terpenes had the function of attracting pollinating insects, which caused the plant to attract a variety of flower visiting insects, and could promote the natural pollination of flowers [32].

There were few reports on the difference in chemical composition of dioecious plants. For example, the contents of 2-cyclohexenone and methyl benzoate in female buds were significantly higher than those in male buds, and the content of ethyl benzoate in male buds was significantly higher than that in female buds of *P. tomentosa* [23]. The content of linalool in female flowers was significantly higher than that in male flowers, whereas the content of benzyl alcohol in male flowers was significantly higher than that in female flowers of *T. kirilowii* [29]. The comparative study on the volatile compounds of the buds revealed the characteristic difference between female and male buds of *H. pedunculosum*, pointed out the preliminary relationship between the volatile compounds and plant physiology, and enriched the basic research of dioecious plants.

Based on the differences of the volatile compounds from female and male buds of *H. pedunculosum*, the synthesis mechanism of these differential compounds and their specific effects on plant growth should be studied in the future. In addition, the differences in non-volatile compounds and biological activities between female and male plants are also worthy of further study.

## 4. Materials and Methods

### 4.1. Apparatus

The SPME fiber 50 mm carboxen/polydimethylsiloxane/divinylbenzene (CAR/PDMS/DVB) was purchased from Supelco (Bellefonte, PA, USA). An AOC-5000 auto injector solid-phase microextraction device and a SHIMADZU QP 2010 gas chromatography–mass spectrometer were purchased from Shimadzu (Tokyo, Japan). An ME204/02 electronic balance was purchased from Mettler Toledo Instruments Co., Ltd. in Shanghai in China.

### 4.2. Sample Collection

Because *H. pedunculosum* was a night-flowering plant, the buds of both female and male plants were collected at 11:00 pm just before they bloomed. The collection date was 31 August 2021, and the collection site was located in the Shangri-La Alpine Botanical Garden, Diqing Tibetan Autonomous Prefecture, Yunnan Province (27°54′17.07″ N, 99°38′15.02″ E, and 3269 m altitude). The female and male buds were obtained by pinching off the flower stalk with tweezers, which were identified as fresh female and male buds of *Herpetospermum pedunculosum* (Ser.) C. B. Clarke (Family Cucurbitaceae) by Prof. Zhimao Chao (Institute of Chinese Materia Medica, China Academy of Chinese Medical Sciences) according to the description in Flora of China (Editorial Board of Flora of China, 1984). The voucher specimens (HPF1-3 and HPM1-3) were deposited at 1022 laboratory of Institute of Chinese Materia Medica, China Academy of Chinese Medical Sciences, Beijing, China.

### 4.3. Sample Preparation

A fresh bud sample was placed in a 15 mL headspace vial. A SPME fiber 50 mm CAR/PDMS/DVB was extended through the needle and exposed into the headspace vial to adsorb volatile compounds at 40 °C for 30 min and then immediately injected into the gas chromatography injection port at 250 °C for 3 min to desorb volatile compounds.

### 4.4. Chromatographic Condition

The volatile compounds from the buds were analyzed by GC-MS method. A GC-MS QP 2010 HP-5MS (SHIMADZU, Tokyo, Japan) was used coupled to UI elastic quartz capillary column (0.25 μm × 0.25 mm × 30 m). The splitless injection mode was used. The carrier gas was high purity helium which was used at a constant flow rate of 1 mL/min. The temperature of injection port was 250 °C. The heating program was as follows: the initial temperature was 40 °C and held for 5 min; the temperature was increased to 190 °C at a rate of 3 °C/min; then increased to 300 °C at a rate of 20 °C/min and held for 1 min.

### 4.5. MS Condition

The ion source temperature was 200 °C. The interface temperature was 230 °C. The solvent delay time was 2 min. The scanning range was *m/z* 45–500. The mass spectra would be obtained at 70 eV with electron ionization (EI) mode.

### 4.6. Data Processing

The mass spectral identification of volatile compounds was obtained by comparing with the National Institute of Standards and Technology (NIST) 14. The qualitative analysis of mass spectral data was verified by comparing the retention time and mass spectra. The quantitative analysis of volatile compounds was determined by peak area normalization of total ion chromatography.

### 4.7. Statistical Analysis

All multivariate analysis and calculations were performed on SIMCA-P software (version 14.1, Umetrics, Malmö, Sweden). The data were imported into the software and scaled by Pareto scaling method to reduce the relative importance of large values and to keep the data structure partially intact. Then, the data were submitted to PCA and OPLS-DA analysis. The differential compounds between female and male buds were filtered and used for the subsequent in-depth analysis.

## 5. Conclusions

In this study, HS-SPME-GC-MS technology coupled to multivariate statistics analysis was used to explore some difference in volatile compounds of *H. pedunculosum.* It was found that there was a significant difference between female and male buds. In total, 38 volatile compounds were identified by GC-MS, and 18 and 20 volatile compounds were detected in female and male buds, respectively. Among them, 6 unique compounds were detected, such as (*E*)-4-hexenol and isoamyl acetate in female buds, 20 unique compounds were detected such as (*Z*)-3-methylbutyraldehyde oxime and benzyl alcohol in male buds. In total, 9 differential volatile compounds of female and male buds were screened out, including isoamyl alcohol, (*Z*)-3-methylbutanal oxime, and 1-nitropentane based on multivariate statistical analysis. This is the first time that the differences in volatile compounds of the dioecious plant *H. pedunculosum* with HS-SPME-GC-MS have been reported, which reveals the essential differences in female and male buds.

## Figures and Tables

**Table 1:** Volatile compounds of GC-MS analysis from female and male buds of *H. pedunculosum.*

No.	Rt/min	Compound	F	CAS	MW	Fragment (*m*/*z*)	Relative Content/%						
							Female			Male			
							1	2	3	1	2	3	
1	4.044	Isoamyl alcohol	C_5_H_12_O	123-51-3	88	70/55/42	90.19	83.29	90.17	43.23	57.90	64.01	
2	8.555	(*E*)-4-Hexenol	C_6_H_12_O	928-92-7	100	82/67/55//41	1.24	0.64	1.92	-	-	-	
3	8.675	(*Z*)-3-Methylbutanal oxime	C_5_H_11_NO	5780-40-5	101	86/59/41	-	-	-	7.15	6.71	6.66	
4	8.960	*p*-Xylene	C_8_H_10_	106-42-3	106	91/76	-	-	-	2.57	2.11	1.83	
5	9.513	3-Methyl-1-butanol acetate	C_7_H_14_O_2_	123-92-2	130	70/55/43	1.66	1.31	1.69	-	-	-	
6	9.438	(*E*)-3-Methylbutanal oxime	C_5_H_11_NO	5775-74-6	101	86/59/41	-	-	-	2.11	2.13	1.94	
7	10.000	*o*-Xylene	C_8_H_10_	95-47-6	106	91/76	-	-	-	0.3	0.15	0.11	
8	10.393	2,2-Dimethyl-1-butanol	C_6_H_14_O	1185-33-7	102	71/43/27	-	-	-	1.38	1.30	1.13	
9	10.578	1-Nitropentane	C_5_H_11_NO_2_	628-05-7	117	71/43/29	-	-	-	5.35	5.39	4.82	
10	11.791	2-Methyl-5-(1-methylethyl)-bicyclo[3.1.0]hex-2-ene	C_10_H_16_	2867-05-2	136	93/77	-	-	-	0.13	0.10	0.07	
11	12.055	1*R*-α-Pinene	C_10_H_16_	7785-70-8	136	121/93/77	0.17	0.29	0.15	0.12	0.11	0.11	
12	13.451	Benzaldehyde	C_7_H_6_O	100-52-7	106	77/51	-	-	-	0.10	0.08	0.07	
13	14.158	Sabinene	C_10_H_16_	3387-41-5	136	93/77/41	0.17	0.33	0.14	1.02	0.53	0.45	
14	15.227	β-Myrcene	C_10_H_16_	123-35-3	136	93/69/41/27	-	-	-	0.61	0.36	0.25	
15	16.423	(+)-4-Carene	C_10_H_16_	29050-33-7	136	121/93/79	-	-	-	0.07	0.06	0.05	
16	16.846	*p*-Cymene	C_10_H_14_	535-77-3	134	119/91	-	-	-	0.17	0.13	0.11	
17	17.044	^TM^*δ*-Limonene	C_10_H_16_	5989-27-5	136	121/107/93/68	1.64	1.95	1.19	2.20	2.95	2.10	
18	17.258	2-Ethyl-1-hexanol	C_8_H_18_O	104-76-7	130	70/57/41/29	1.16	2.86	1.61	-	-	-	
19	17.351	Benzyl alcohol	C_7_H_8_O	100-51-6	108	91/79/77/51	-	-	-	1.17	0.81	0.86	
20	17.695	α-Pinene	C_10_H_16_	80-56-8	136	93/77/39/27	-	-	-	0.50	0.21	0.10	
21	18.209	β-Ocimene	C_10_H_16_	13877-91-3	136	93/79/53/39	1.19	0.56	0.79	5.69	5.22	5.58	
22	18.638	γ-Terpinene	C_10_H_16_	99-85-4	136	121/93/77/43	0.19	0.33	0.15	0.65	0.39	0.26	
23	21.073	Nonanal	C_9_H_18_O	124-19-6	142	98/70/57/41	0.15	0.39	0.15	0.15	0.14	0.14	
24	22.362	2-Methyl-1-undecene	C_12_H_24_	18516-37-5	168	69/56/41	-	-	-	1.36	1.70	1.08	
25	22.934	4-Oxoisophorone	C_9_H_12_O_2_	1125-21-9	152	96/68/39	-	-	-	5.57	5.79	5.62	
26	24.110	2,2,6-Trimethyl-1,4-cyclohexanedione	C_9_H_14_O_2_	20547-99-3	154	139/69/56/42	-	-	-	2.68	1.97	1.88	
27	30.453	*n*-Pentadecane	C_15_H_32_	629-62-9	212	85/71/57/43	0.19	0.27	0.19	-	-	-	
28	33.704	α-Copaene	C_10_H_16_	3856-25-5	136	161/119/105/91	-	-	-	0.09	0.10	0.11	
29	34.834	*n*-Hexadecane	C_14_H_30_	629-73-2	198	85/57/43	0.35	0.51	0.22	-	-	-	
30	35.068	(+)-7-epi-Sesquithujene	C_15_H_24_	159407-35-9	204	119/93/77/69/41	0.13	0.13	0.13	0.50	0.88	0.97	
31	35.435	trans-α-Bergamotene	C_15_H_24_	13474-59-4	204	119/107/93/69/41	0.25	0.13	0.17	1.11	1.94	2.17	
32	37.199	(1*S*,5*S*)-4-Methylene-1-((*R*)-6-methylhept-5-en-2-yl)bicyclo[3.1.0]hexane	C_15_H_24_	58319-04-3	204	93/69/41	-	-	-	1.03	1.71	1.85	
33	39.901	(1*R*,5*R*)-4-Methylene-1-((*R*)-6-methylhept-5-en-2-yl)bicyclo[3.1.0]hexane	C_15_H_24_	58319-04-3	204	93/69/41	0.64	0.45	0.57	1.39	1.78	1.99	
34	51.476	Neophytadiene	C_20_H_38_	504-96-1	278	123/95/82/68/55/41	0.27	0.76	0.35	-	-	-	
35	54.139	(*E*,*E*,*E*)-3,7,11,15-Tetramethylhexadeca-1,3,6,10,14-pentaene	C_20_H_32_	77898-97-6	272	93/81/69/55/41	-	-	-	0.10	0.10	0.10	
36	56.232	(*E*,*E*)-7,11,15-Trimethyl-3-methylene-1,6,10,14-hexadecatetrene	C_20_H_32_	70901-63-2	272	93/81/69/41	0.21	0.67	0.17	0.24	0.14	0.13	
37	57.391	(*Z*)-9-Tricosene	C_23_H_46_	27519-02-4	322	97/83/55	-	-	-	0.14	0.14	0.18	
38	57.569	*n*-Eicosane	C_20_H_42_	112-95-8	282	85/71/57/43	0.21	0.13	0.23	0.12	0.18	0.26	

F: formula; MW: molecular weight; -: not be detected.

**Table 2:** Nine differential volatile compounds between female and male buds of *H. pedunculosum.*

Section	No.	Compound	VIP
A	1	Isoamyl alcohol	4.00
B	3	(*Z*)-3-Methylbutanal oxime	1.83
B	9	1-Nitropentane	1.59
B	21	β-Ocimene	1.48
B	25	4-Oxoisophorone	1.26
B	24	2-Methyl-1-undecene	1.07
B	26	2,2,6-Trimethyl-1,4-cyclohexanedione	1.03
B	4	*p*-Xylene	1.03
B	6	(*E*)-3-Methylbutanal oxime	1.00

A: higher in females than in males; B: higher in males than in females.

## Data Availability

All the relevant data have been provided in the manuscript. The authors will provide additional details if required.

## Sample Availability

Not available.
